# The REFRACT-LYMA cohort study: a French observational prospective cohort study of patients with mantle cell lymphoma

**DOI:** 10.1186/s12885-016-2844-6

**Published:** 2016-10-14

**Authors:** Matthieu Hanf, David Chiron, Sophie de Visme, Cyrille Touzeau, Hervé Maisonneuve, Henry Jardel, Catherine Pellat-Deceunynck, Martine Amiot, Steven le Gouill

**Affiliations:** 1 INSERM CIC 1413, Centre Hospitalier Universitaire de Nantes, Nantes, France; 2 INSERM, UMR892 - CNRS, UMR 6299, Université de Nantes, Nantes, France; 3 Service d’Hématologie Clinique, Unité d’Investigation Clinique, Centre Hospitalier Universitaire de Nantes, Nantes, France; 4 Service de Médecine Onco-hématologie, Centre Hospitalier Départemental de La Roche sur Yon, La Roche sur Yon, France; 5 Service de Médecine interne - Maladies hématologiques - Maladies infectieuses, Centre Hospitalier Bretagne Atlantique, Vannes, France; 6 CHU de Nantes, Place Alexis Ricordeau, 44000 Nantes, France

**Keywords:** Cancer, Mantle cell lymphoma, Cohort, Therapeutic failure, Biological samples, Functional assays, Quality of life, Epidemiology, Pharmacoeconomics

## Abstract

**Background:**

Mantle Cell Lymphoma (MCL) is often associated with progression, temporary response to therapy and a high relapse rate over time resulting in a poor long-term prognosis. Because MCL is classified as an incurable disease, therapeutic resistance is of great interest. However, knowledge about the biological mechanisms underlying resistance associated with MCL therapies and about associated predictors remains poor. The REFRACT-LYMA Cohort, a multicenter prospective cohort of patients with MCL, is set up to address this limitation. We here describe the study background, design and methods used for this cohort.

**Methods/Design:**

The REFRACT-LYMA Cohort Study aims at including all patients (>18 years old) who are diagnosed with MCL in any stage of the disease and treated in specialized oncology centers in three public hospitals in Northwestern France. Any such patient providing a signed informed consent is included. All subjects are followed up indefinitely, until refusal to participate in the study, emigration or death. The REFRACT-LYMA follow-up is continuous and collects data on socio-economic status, medical status, MCL therapies and associated events (resistance, side effects). Participants also complete standardized quality of life (QOL) questionnaires. In addition, participants are asked to donate blood samples that will support ex vivo analysis of expression and functional assays required to uncover predictive biomarkers and companion diagnostics. If diagnostic biopsies are performed during the course of the disease, extracted biological samples are kept in a dedicated biobank.

**Discussion:**

To our knowledge, the REFRACT-LYMA Cohort Study is the first prospective cohort of patients with MCL for whom “real-life” medical, epidemiological and QOL data is repeatedly collected together with biological samples during the course of the disease. The integrative cohort at mid-term will be unique at producing a large variety of data that can be used to conceive the most effective personalized therapy for MCL patients. Additionally, the REFRACT-LYMA Cohort puts the medical care of MCL patients in a health and pharmacoeconomic perspective.

## Background

Mantle Cell Lymphoma (MCL) is a Non-Hodgkin lymphoma (NHL) defined as a unique type of B-cell lymphoma entity [1]. MCL accounts for approximately 5–10 % of all NHLs. Its incidence is estimated at around 0.5 per 100,000 person/year [2, 3]. Patients are generally Caucasian, male and elderly [2]. Some data suggests that MCL incidence may vary geographically and may possibly have increased over the last decades [2]. While some NHLs have been found to be related to specific inherited, environmental or infectious exposures, no strong and consistent relationship has been established for MCL [2, 4]. There are indeed but few studies of potential risk factors, often retrospective and primarily conducted by pooling all NHLs. However, it has been suggested that MCL might be associated with exposure to *Borrelia burgdoferi* [5] and with family history of hematopoietic malignancies and of genetic variation in the pro-inflammatory cytokine interleukin 10 [6, 7]. All these findings must be confirmed and remain controversial. Large prospective studies with sufficient statistical power and data quality are needed to confirm/discover risk factors associated with MCL.

MCL is usually diagnosed as a late-stage disease that has typically spread to the gastrointestinal tract and bone marrow [8]. MCL is often associated with temporary response to therapy, varying from months to decades, and the high relapse rate over time results in a poor long-term prognosis [9]. Overall survival (OS) is heterogeneous with a reported median around 5 years [3, 10]. An efficient prognostic index has been developed to predict OS: the Mantle Cell Lymphoma International Prognostic Index (MIPI) [11]. It classifies patients into three risk groups: low, intermediate and high risk. However, as emphasized elsewhere [4], the MIPI is prognostic for survival, not for therapeutic decisions and has been only validated for first-line therapy. Additional research is needed to identify associated risk factors and develop appropriate prognostic scores for all these situations.

Because MCL is classified as an incurable disease, therapeutic resistance is of great interest. In MCL, therapeutic resistance may schematically be divided in two categories: 1) primary resistance (the disease does not respond to the therapeutic agent(s) because of intrinsic characteristics and/or of a protective environment) and 2) acquired resistance (the disease was sensitive but relapse occurs as one or several resistant subclones emerge, that initially were in minority or that were acquired through therapeutic pressure) [12]. The Darwinian selective pressure and associated biological mechanisms still need to be clarified. This is crucial in order to decide whether to establish a therapy and/or to extend it or not. Biobanks of tumor samples, collected in a standardized manner before launching new therapies and at potential therapeutic failures, are required to achieve these goals.

Furthermore, MCL therapies have a huge economic impact on society. Temsirolimus, the first drug to receive EMA approval, costs £36,000 per year [13]. Estimates say that an Ibrutinib therapy (70 % of respondent patients in monotherapy) costs $110,000 per year in the United States [14]. It is strategic to identify predictors of response/sensitivity to therapies precisely. This would not only spare patients unnecessary therapies, but also optimize healthcare resources and reduce unnecessary costs. The issue is reinforced by growing availability of other promising molecules, e.g. ABT-199 (GDC-199), BTK/PI3K inhibitors or new antibodies [15–18]. Possessing a cohort of MCL patients is a strategic asset for the pharmacoeconomic evaluation of associated therapies.

Finally, Quality of life (QOL) is recognized by clinicians and researchers to be an important indicator for cancer patients, both in clinical management and for the cost-benefit evaluation of therapies. Various studies of the impact of QOL on hematological cancers and associated therapies [19] have been performed. There have been fewer studies of NHL and even less of MCL. A recent study shows that QOL should be assessed at diagnosis and used as prognostic factor in patients with aggressive lymphoma [20]. Some studies focus on the QOL of long-term survivors of non-Hodgkin lymphoma [21]. Measuring QOL is necessary to get an integrative vision of MCL and to improve clinical management.

To address all these limitations, we have developed a multicenter prospective cohort of patients with MCL: the REFRACT-LYMA Cohort.

### Study objectives

The goals of the REFRACT-LYMA Cohort Study are:

- To improve the characterization of MCL epidemiology (incidence, temporal trends, geographical disparities, risk factors, nature of treatments, duration of response to each sequential line of treatment)
- To understand the biological mechanisms that precipitate the occurrence of severe side effects, response or resistance associated to MCL therapies
- To identify new therapeutic targets for the MCL treatment
- To determine the clinical, biological and quality of life impacts of MCL therapies
- To evaluate the temporal dynamic of patient response to therapies
- To identify patient characteristics affecting primary resistance development or severe side effects associated with MCL therapies
- To find and optimize the most effective therapeutic associations in MCL treatment
- To construct reliable prognostic scores predicting response to MCL therapies
- To conduct pharmaco-economic evaluations of MCL therapies
- To develop evidence-based therapeutic algorithms based on biological characteristics of a patient's tumor
- To further basic research in the field of MCL

The focus of this report is describing the methodology used to establish and manage this cohort.

This program is based on a network that includes the Medical university of Nantes, the INSERM_U892 CNRS_U6299 research unit (Cancer Research Center Nantes-Angers, Nantes), the Nantes university Hospital and the General Hospitals in La Roche sur Yon and in Vannes. All centers follow the same treatment guideline. Fit untreated patients below the age of 70y are treated with a cytarabine-based plus rituximab chemotherapy regimen followed by autologous stem cell transplantation and rituximab maintenance, while elderly or unfit patients receive R-CHOP plus Rituximab maintenance. According to various paraeters (duration of response, age, fit/unfit..), patients are commonly treated with a bendamustine-based chemotherapy regimen. Allogeneic-stem cell transplantation is discussed for refractory young patients. Ibrutinib is offered to relapse refractory patients and inclusion in clinical trials can also be proposed.

## Design

### Study design

The REFRACT-LYMA Study is an observational cohort study that aims at including all patients diagnosed with MCL followed in specialized oncology centers within three public hospitals in Northwestern France (Nantes University Hospital and the General Hospitals in La Roche sur Yon and in Vannes) over at least a 10-year period. All newly diagnosed patients are followed prospectively. Patients already dealt with in study sites at study start are followed prospectively, from their inclusion and onwards. For these patients, previous clinical data is recorded retrospectively from medical files. The cohort is registered by the French Data Protection Authority in clinical research (Commission Nationale de l’Informatique et des Libertés or CNIL). The study was approved by an ethics committee (Groupe Nantais d'Ethique dans le Domaine de la Santé, RC14_0358).

### Study population

Adult men and women (>18 years old) diagnosed with MCL according to the 2008 World Health Organization classification of lymphoid neoplasms [1] in any stage of the disease at any of the three study hospitals are eligible for the study. The study excludes non French-speaking patients with no social insurance and patients with dementia or mental conditions that prevent them from completing the surveys correctly. The physicians recruit the patients. Once they have been informed that participating is voluntary and anonymous, all patients who give informed consent are included. The study physicians ask all participants if they have properly understood the study.

### Data collection

The REFRACT-LYMA study is based on a continuous and comprehensive record of pre-specified and standardized information on all included patients.

Specific data is compiled at baseline plus at each follow-up visit, according to the reason for follow-up. In the REFRACT-LYMA Cohort Study, the reason for follow-up is categorized as follows: disease diagnostic, disease progression, disease relapse, treatment initiation, treatment monitoring, treatment toxicity, end of treatment and monitoring in absence of treatment. As the study is non-interventional, each study physician follows included patients as well as possible according to their clinical, biological, psychological and social situation. Subjects are followed indefinitely until secondary refusal to take part in the study, emigration or death. Save for a significant event (therapeutic failure, adverse events…), the three study hospitals will usually follow up patients every 6 months.

Follow-up is continuous. It includes collecting data on socioeconomic and medical status plus on MCL therapies and associated events (resistance and side effects). The participants also complete standardized quality of life questionnaires. The participants are additionally asked to give a blood sample at baseline and at specific follow-up stages. For any diagnostic biopsy performed during the course of the disease (e.g. bone marrow, lymph node, pleural effusion, cerebrospinal fluid), extracted biological samples will also be kept in a dedicated biobank. Table 1 shows the collected data according to reason for follow-up throughout the study.

**Table 1:** Collected data throughout the REFRACT-LYMA cohort study

Collected data	Reason for follow-up							
	Baseline visit	Monitoring without treatment	Monitoring with treatment	Disease progression	Disease Relapse	New treatment	Treatment toxicity	End of treatment
Sociodemographic data								
Age	X							
Sex	X							
Education	X							
Profession^a^	X							
Living situation^a^	X							
Place of residence^a^	X							
Ethnicity	X							
Number of children^a^	X							
Death information^a^								
Epidemiological data								
Body weight and height^a^	X							
Physical activities^a^	X							
Smoking and alcohol habits^a^	X							
Family history of cancer	X							
Personal history of lymphoma	X							
Constitutive or acquired immunodeficiency	X							
Medical data								
ECOG performance status	X	X	X	X	X	X	X	X
Disease status	X	X	X	X	X	X	X	X
Ann Arbor staging	X		X	X	X	X		
IPI	X		X	X	X	X		
MIPI	X		X	X	X	X		
Splenomegaly	X		X	X	X	X		
Digestive expression of lymphoma	X		X	X	X	X		
Circulating tumor cells	X		X	X	X	X		
Number of extranodal territories	X		X	X	X	X		
Serum lactate dehydrogenase level	X		X	X	X	X		
Creatinine level	X		X	X	X	X		
Hemoglobin concentration	X		X	X	X	X		
White blood cell count	X		X	X	X	X		
Bone marrow biopsy data	X		X	X	X	X		
Tumor cell cytological morphology	X		X	X	X	X		
Molecular markers	X		X	X	X	X		
Genetic abnormality: t(11,14) and del17	X		X	X	X	X		
B-cell and MCL markers	X		X	X	X	X		
Monoclonality	X		X	X	X	X		
MRD	X	X	X	X	X	X		
PET data	X		X	X	X	X		
Adverse events	X	X	X	X	X	X	X	X
Treatment data								
Treatment characteristics	X		X	X	X	X		X
Comorbidity								
CIRS-G^a^	X							
Quality of life	X	X	X	X	X	X	X	X
Biological samples and functional assays								
Biological samples: blood sample, bone marrow, lymph node, pleural effusion, cerebrospinal fluid	X		X	X	X	X	X	
Flow cytometry	X		X	X	X	X	X	
RNA analysis:	X		X	X	X	X	X	
Functional assays	X		X	X	X	X	X	

^a^Data updated every year

### Sociodemographic and epidemiological data

Sociodemographic and epidemiological characteristics are assessed by means of standardized questionnaires managed by the physicians. They cover demographics (birth year, sex, education, profession, living situation, place of residence, ethnicity, number of children and, when applicable, date and associated cause of death), body weight, height, physical activities, smoking and alcohol habits, family and personal history of lymphoma and others types of cancer and of constitutive or acquired immunodeficiency. This questionnaire is completed at baseline and follow-up. Time-varying sociodemographic and epidemiological data is updated every year.

### Medical and therapeutic data

The Eastern Cooperative Oncology Group (ECOG) Performance Status [22] and disease status (complete remission/stable disease/partial response) are systematically assessed at follow-up. The International Prognostic Index (IPI) [23] and MIPI [11] calculation results are calculated. The treatment initiation date, treatment type, response to treatment and date of response are recorded for each administrated treatment.

The REFRACT-LYMA Cohort also collects the Ann Arbor staging (I, II, III, IV), the presence/absence of splenomegaly, a digestive expression of lymphoma and circulating tumor cells, the number of extranodal territories, the serum lactate dehydrogenase and creatinine levels, the hemoglobin concentration and the white blood cell count, as needed for the IPI [23] and the MIPI [11] calculation.

Anatomological and genetic characteristics are assessed with data from a bone marrow biopsy with associated results (absence/presence of bone marrow lesions). Tumor cells are classified into classic or variant types (blastoid variant and polymorphic variant) according to cytologic morphology. Information on specific molecular markers and on t(11,14) genetic translocation or del17 genetic deletion is recorded. Quantitative data on expressions of B-cell and MCL markers and on the Minimal Residual Disease (MRD) is collected. If positron emission topography (PET) is used during follow-up, the date, place and associated standardized uptake values are registered.

### Adverse event data

All adverse events with date of occurrence are recorded in the database. The classification used to describe the severity of organ toxicity for patients receiving cancer therapy [24] is based on the Common Terminology Criteria for Adverse Events (CTCAE) V3.0 from the National Cancer Institute (NCI) of the National Institutes of Health (NIH). Toxicity is graded as mild (Grade 1), moderate (Grade 2), severe (Grade 3) or life-threatening (Grade 4), with specific parameters according to the organ system involved. Death (Grade 5) is used for some criteria to point out fatality. In this shared terminology, adverse event means any abnormal clinical finding temporally associated with the use of a therapy. The causality is not required.

### Comorbidity

Comorbidity is recorded with the Cumulative Illness Rating Scale for Geriatrics (CIRS-G) [25]. This scoring system addresses 14 organ systems (vascular problems, hematopoietic system, respiratory tract, liver, gallbladder, pancreas, endocrine and metabolic disease, breast, heart, musculoskeletal/integumentary, lower gastrointestinal tract, eyes, ears, nose, throat, genitourinary tract, upper gastrointestinal tract, neurological disease, renal disorder and psychiatric disorder). For each patient, comorbidity is associated with an organ system and rated from one (mild comorbidity) to four (extremely severe comorbidity). If more than one disease occur in the same organ system, only the most severe is rated. If a disease can be traced back to the primary disorder (the reason for hospitalization), it is not recorded as comorbidity. This questionnaire is completed at baseline and follow-up. Associated data is updated every year.

### Quality of life

Three measures are used to assess quality of life in patients with Mantle cell lymphoma at baseline and follow-up.

First, the Medical Outcomes Study Short Form (SF-36) survey, a general physical and mental health measure, is used for comparison with general population norms. It covers 36 items organized into eight subscales and two summary scores, the Physical Component Score (PCS; Physical Functioning, Role-Physical, Bodily Pain and General Health) and Mental Component Score (MCS; Vitality, Social Functioning, Role-Emotional and Mental Health) [26].

Second, the EuroQOL five dimensions survey (EQ-5D) facilitates pharmacoeconomic evaluation. It is a standardized instrument measuring health-related quality of life and applicable to a wide range of health conditions and treatments [27]. It is a simple questionnaire with five questions and a visual analogue scale (EQ-VAS). EQ-5D is primarily designed for self-completion by respondents and ideally suited for postal surveys, in clinics or during face-to-face interviews. It has been developed and validated in many countries, including France.

Third, the Functional Assessment of Chronic Illness Therapy - Lymphoma (FACT-Lym) is used to capture lymphoma-specific QOL [28]. This short, adequately validated QOL measure consists of a general module (FACT-G), four subscales (physical, social/family, emotional and functional) and a 15-item lymphoma specific module listing lymphoma-related symptoms (e.g. fever, night sweats, itching).

### Biological samples and functional assays

Blood samples are collected from the patients upon recruitment and at specific follow-up appointments (Table 1). For each blood sample and immediately after blood draw, specific biomarkers are assessed by flow cytometry. In addition to classical analysis related to medical data, cytokine receptors such as CD210 (IL10R), CD221 (IGF1R), CD126 (IL6R), CD360 (IL21R), CD268 (BAFFR), extracellular markers such as CD38, CD40, CD138, and viability (Annexin-V) are addressed. After purification using MACS CD19 MicroBeads (Miltenyi Biotec), the percentage of MCL tumor cells exceeds 90 %, measured by flow cytometry. Some purified cells are stored as dry pellets for further RNA, DNA or protein analysis, others are used for functional assays. RNA analysis includes systematic PCR quantification of genes previously described as related to chemoresistance such as Bcl-2, Mcl-1, Bcl-xL or Bim (*BCL2L11*) (ref 18). Several markers of MCL aggressive behavior, e.g. Ki67 and Sox11 [29], are also determined. We will also assess the integrity of the p53 pathway, chromosomal deletion and/or somatic mutations that have been reported as prognosis markers [30].

Ex vivo functional assays and correlation with targeted therapy response are matched with expression analysis, to develop predictive biomarkers and companion diagnostics. In addition to response at the cellular level, targeted therapy efficacy are matched at the mitochondrial level using functional BH3-profiling [31].

There is growing evidence suggesting a critical role of microenvironment in MCL [32]. Cell cycle, survival, migration and chemoresistance of MCL primary cells are addressed in various relevant coculture models (mesenchymal stromal, lymphoid, myeloid) in presence of MCL specific growth factors. This strategy will document the so far unknown bidirectional communications between MCL primary cells and their surrounding ecosystem. Finally, the main results will be validated in vivo, using relevant mouse models [33].

Remaining blood samples are stored in a unique biobank at −80 °C that is hosted by the INSERM_U892 CNRS_U6299 research unit (CRCNA, Nantes). They will be used later for functional assays and for gene and biomarker analysis. When possible, biopsies from lymph nodes, bone marrow and pleural effusion are analyzed using the same protocol as for the blood samples.

### Data management

This multicenter study relies on a web-based system with data being entered into a central database. The system provides security with a protected access and complies with French safety policy. For each new patient included and associated follow-up, data is collected on paper forms and entered into the web-based data entry portal by the project workers and the medical team. The data management team performs a data quality control every year. The local medical team is notified in case of discrepancy or incomplete data.

### Sample size and statistical analysis

In this study, the number of patients coming to the three study hospitals to consult for MCL per year determines the cohort size. All patients accepting to participate are included. Altogether around 50 patients diagnosed with MCL are taken in by the three study oncology centers every year. Roughly half of these are new. Based on the study physicians’ experience, 10 % of patients refuse to participate or don’t meet the inclusion criteria. A final sample size of around 240 patients is thus expected for a study period of 10 years including interim analyses performed at 3 and 5 years.

Confidence intervals, means, standard deviations and frequency distributions are calculated for all measures. Corrections for multiple comparisons are used to control the occurrence of type I statistical errors. All analyses are performed with R [34]. Kaplan-Meier survival curves are used to compare follow-up data. Logistic regression or Cox regression adjusts for patient characteristics and is used for survival analysis for different groups of patients. Depending on the statistical power available, various advanced statistical models are used.

## Discussion

To our knowledge, this is the first prospective cohort of patients with MCL where medical, epidemiological and QOL data is repeatedly collected in combination with biological samples collected before treatment initiation and at treatment failure. Recruitment is expected to start by January 2016. In the mid-term, this integrative cohort will be a unique data source enabling each MCL patient to conceive the most effective personal therapy according to a large variety of data (patient tumor biological characteristics, epidemiological data, patient medical history, QOL measures and ex-vivo functional assays). Additionally, the REFRACT-LYMA Cohort will answer health and pharmacoeconomic questions about the medical care of MCL patients using “real-life data”, as opposed to clinical trials. However, larger studies may be necessary for these areas to be investigated in depth and to allow for solid and reproducible conclusions. That is why the REFRACT-LYMA Cohort uses standardized data, facilitating future collaborative data pooling as far as possible. The REFRACT-LYMA Cohort is also designed to facilitate collaboration with investigators, institutions, cooperative groups and pharmaceutical companies working in the MCL area.

## Abbreviations

CIRS-G: Cumulative Illness Rating Scale for Geriatrics
CTCAE: Common Terminology Criteria for Adverse Events
FACT-Lym: Functional Assessment of Chronic Illness Therapy - Lymphoma
IPI: The International Prognostic Index
MCL: Mantle cell lymphoma
MCS: Mental component score
MIPI: Mantle Cell Lymphoma International Prognostic Index
MRD: Minimal residual disease
NCI: National Cancer Institute
NHL: Non-Hodgkin lymphoma
NIH: National Institutes of Health
PCS: Physical component score
PET: Positron emission topography
